# Hosts, microbiomes, and the evolution of critical windows

**DOI:** 10.1002/evl3.298

**Published:** 2022-10-27

**Authors:** C. Jessica E. Metcalf, Burcu Tepekule, Marjolein Bruijning, Britt Koskella

**Affiliations:** ^1^ Department of Ecology and Evolutionary Princeton University Princeton New Jersey 08544; ^2^ Wissenschaftskolleg zu Berlin DE‐14193 Berlin Germany; ^3^ Department of Integrative Biology UC Berkeley Berkeley California 94720

**Keywords:** Critical window, host evolution, microbiome

## Abstract

The absence of microbial exposure early in life leaves individuals vulnerable to immune overreaction later in life, manifesting as immunopathology, autoimmunity, or allergies. A key factor is thought to be a “critical window” during which the host's immune system can “learn” tolerance, and beyond which learning is no longer possible. Animal models indicate that many mechanisms have evolved to enable critical windows, and that their time limits are distinct and consistent. Such a variety of mechanisms, and precision in their manifestation suggest the outcome of strong evolutionary selection. To strengthen our understanding of critical windows, we explore their underlying evolutionary ecology using models encompassing demographic and epidemiological transitions, identifying the length of the critical window that would maximize fitness in different environments. We characterize how direct effects of microbes on host mortality, but also indirect effects via microbial ecology, will drive the optimal length of the critical window. We find that indirect effects such as magnitude of transmission, duration of infection, rates of reinfection, vertical transmission, host demography, and seasonality in transmission all have the effect of redistributing the timing and/or likelihood of encounters with microbial taxa across age, and thus increasing or decreasing the optimal length of the critical window. Declining microbial population abundance and diversity are predicted to result in increases in immune dysfunction later in life. We also make predictions for the length of the critical window across different taxa and environments. Overall, our modeling efforts demonstrate how critical windows will be impacted over evolution as a function of both host‐microbiome/pathogen interactions and dispersal, raising central questions about potential mismatches between these evolved systems and the current loss of microbial diversity and/or increases in infectious disease.

**Impact Summary**

The study of host‐microbe coevolution is a bedrock of evolutionary biology. Although microbes have long consumed much of the attention, features of host immune system function are increasingly emerging that call for evolutionary explanation. Here, we focus on the question of the existence of a critical window in immune function, lately identified as a potentially important contributor to microbiome homeostasis, and late age immune dysfunction. If hosts are not exposed to commensal microbes during this critical window, their immune system may not learn to tolerate these microbes, and hosts may then be subject to late‐age immune dysfunction and immunopathology on later encountering them. In addition, exposure to pathogenic microbes, either within the critical window or afterward, will shape the costs and benefits of having a critical window. We explore how features of the ecology of commensal and pathogenic microbes shape selection on the length of this critical window, and discuss our predictions for host species across the tree of life, and in the context of declining microbial diversity and abundance in human populations.

Recent growth in the global burden of immune dysfunction (Devereux 2006; Prescott 2013; Rook et al. 2017; Stiemsma and Turvey 2017) brings urgency to efforts to disentangle contributing factors. The immune system is particularly susceptible to long‐term programming (Kotas and Medzhitov 2015). Early life experiences can reduce or amplify later life inflammatory pathologies (Scharschmidt et al. 2015; Knoop et al. 2017; Davenport et al. 2020; Gerardo et al. 2020; Lynch et al. 2020; Ansaldo et al. 2021; Brodin 2022). Many lines of evidence point to the existence of a “critical window,” during which the immune system can be “trained” to tolerate particular microbes, and thus avoid later destructive immunopathology to these same microbes (Gensollen et al. 2016; Stiemsma and Turvey 2017; Brodin 2022). This design is likely to have evolved because it enables hosts’ immune systems to learn to tolerate commensal microbes that they are likely to encounter later in life. The alternative strategy of generalized tolerance of microbes across life would be vulnerable to exploitation by pathogens. Conversely, generalized immune responsiveness across the life cycle carries a risk because so much of the immune response is dangerous (Graham et al. 2005). Thus, early “education” as to what microbes can be safely ignored is likely to increase host fitness. Importantly, a “critical window” design does require that conditions of the hosts’ early life are predictive of the microbes it will encounter later in life.

That the predictability required by the critical window design is largely reliable is suggested by the fact that animal models have revealed a range of mechanisms associated with “training” the immune system early in life to avoid destructive immunopathology. For example, relative to adult T cells, neonatal T cells preferentially develop tolerance in response to antigen exposure (Gensollen et al. 2016) and antigen‐specific regulatory T cells activated early in life result in tolerance to these same antigens in later life (Scharschmidt et al. 2015). Moreover, Mucosal Associated Invariant T cells (MAIT cells) that create a tolerant immune environment are known to recruit to tissue surfaces in the presence of microbial species, but are outcompeted if the microbial species are not present early in life (Constantinides et al. 2019). The end of the window is also consistently sharp—exposure to microbes beyond a specific timepoint during ontogeny has repeatedly been shown to be incapable of rescuing developing offspring from future immunopathologies (Scharschmidt et al. 2015; Gensollen et al. 2016; Knoop et al. 2017; Constantinides et al. 2019). Both the range of mechanisms that have evolved to result in a “critical window” and the sharpness with which the window ends suggest that strong selective pressures maintain critical windows. Thus, characterizing how selection pressures emerging from microbe and host ecology shape the evolutionarily optimal length of the critical window will provide a lens onto this important aspect of our health.

Framing this problem requires the development of quantitative expressions for how reducing later life overreaction to neutral (or even beneficial) microbes (here termed “commensal” microbes) will increase fitness (Graham, Allen, and Read 2005), but also how the benefits of the reduction of immunopathology come with costs associated with the fact that the immune system plays a primary role in protection against pathogens. Critical windows create an opportunity for the host's immune system to learn to tolerate microbes, but come with a cost of both immediate and long‐term risks associated with pathogen infection. First, pathogens could slip through immune defenses during the phase over which tolerance is acquired; and second, later in life, pathogen antigens experienced during the window may be inappropriately tolerated by the host immune system, allowing greater pathogen growth and thus damage.

The balance of costs and benefits of critical window length will be defined first by direct microbial impacts, that is, the morbidity and mortality burden associated with failure to tolerate commensal microbes, and inappropriate tolerance of pathogens both during the window and subsequently (Fig. 1A). However, considering these direct costs alone will not be sufficient. Microbial ecology will also shape optimal critical window length because microbial ecology determines both the overall prevalence and age of microbial encounters, and the balance of infection/colonization across age will play a critical part in determining the optimal strategy. All else equal, less transmissible microbial taxa will be associated with a higher age of infection and a lower prevalence in the population (Anderson and May 1992). Thus, for example, commensal microbes that are less transmissible will select for a longer critical window, because hosts will have a reduced probability of being infected and thus developing tolerance early in life. Yet, this window must also not be so long as to create too much opportunity for pathogen infection. Additionally, maintaining the window open itself may have a cost. Duration of infection and risks of reinfection following recovery will also shape prevalence and age of infection, as will host demography and seasonality in transmission. All of these features thus have the potential to affect the optimal length of critical windows.

**Figure 1:**
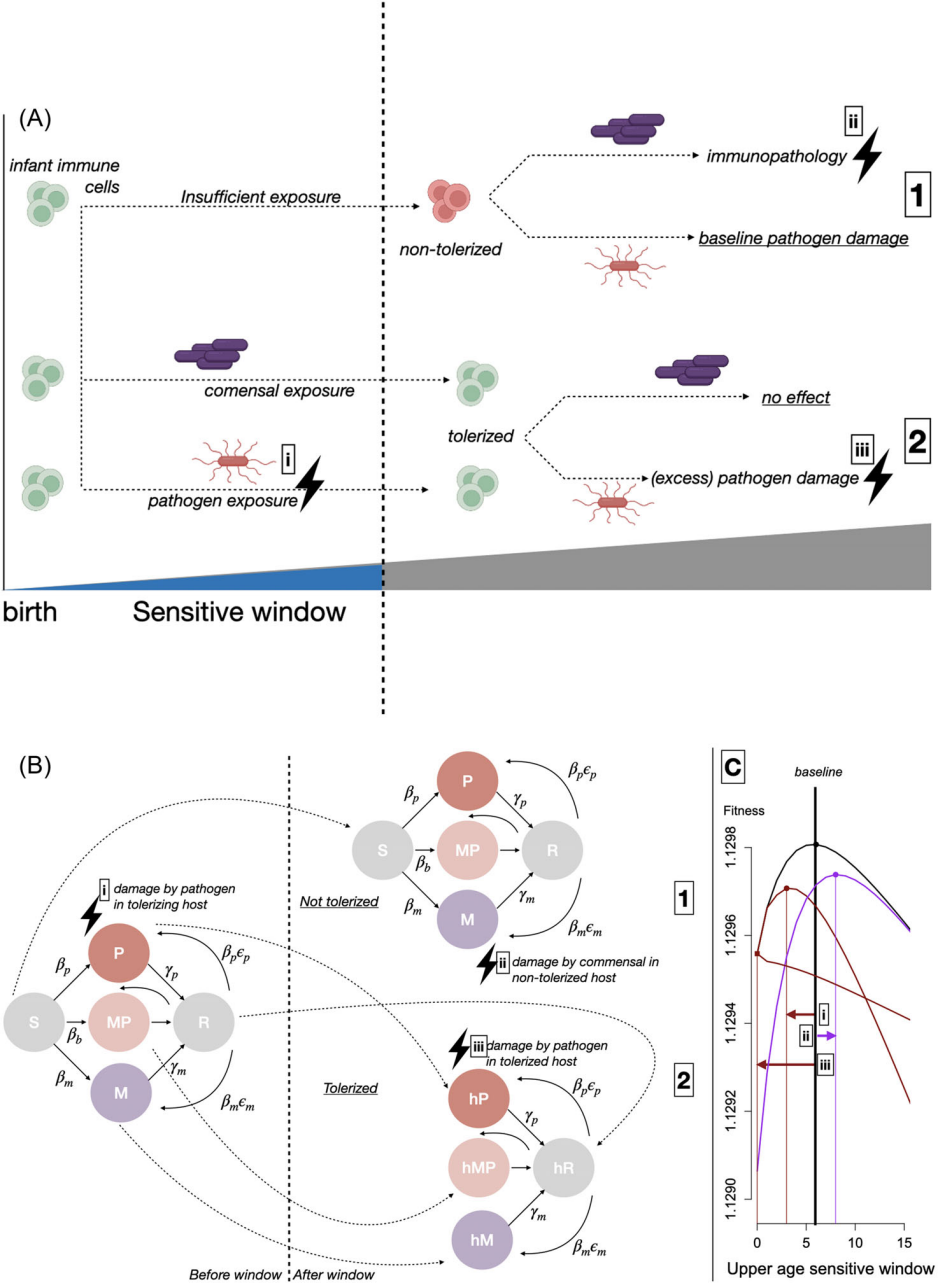
(A) Life course trajectory showing infant immune cells (green) where no infection occurred during the critical window (above, labeled 1) and where it did (below, labeled 2) by a commensal (purple) or a pathogen (red); lightning bolts indicate opportunities for excess mortality. (B) Corresponding life cycle graph. Individuals may be susceptible (*S*), infected by a pathogen (*P*) or commensals (*M*), both together (*MP*), and recovered from both pathogen and commensals (R), with waning of immunity allowing reinfection by commensals or pathogen. Individuals who have remained susceptible during the critical window do not acquire tolerance (above, labeled 1) with higher mortality associated with (co)infection by the commensal; conversely, individuals who were infected (below, labeled 2, and cateogries marked with an h) have higher mortality associated with (co)infection by the pathogen; excess mortality is indicated by the lightning bolt numbered as on the previous panel. For clarity, demographic transitions have been left out, including births into the susceptible class (*S*), or the infected classes (*P* or *M*), depending on the degree of vertical transmission (inheritance) of microbes. Mortality during the critical window is also increased to reflect costs of immune development (see *Methods*). (c) Fitness landscape showing impacts of doubling the three highlighted mortality processes (lightning bolts) relative to a baseline (parameters shown in Table 1). Doubling mortality associated with pathogen infection in a host during the window (i) reduces optimal window length W*, thus reducing risk of infection during the window; doubling mortality associated with commensal infection in a host that has not acquired tolerance (ii) increases it to increase potential for infection and thus acquiring tolerance; and doubling mortality associated with pathogen infection in a host that has acquired tolerance (iii) increases optimal window length to reduce time during which pathogen reinfection could occur.

The experimental design required to establish the optimal length of the critical window would be arduous, complicated, and time consuming. We therefore turn to a mathematical model to characterize the length of the critical window that best allows hosts to balance the competing needs of avoiding excessive immune response to commensal (and ubiquitous) microbes, whereas still maintaining adequate protection against pathogens. This requires developing a mathematical model encompassing host demography as well as microbial transmission. Here, we build such a model and use it to address the question of the optimal length of the critical window across a spectrum of features of microbial biology and associated costs for the host, considering a range of host life histories.

Although our primary focus is on the evolution of a static critical window, critical windows could also be plastic (responsive to the local environment). For example, the microbial context experienced by mothers, or experienced by offspring very early in life, could modulate the presence of the window. Indeed, experiments in mice suggest that pathogen infection of mothers while offspring are in utero increases antipathogen responses (Lim et al. 2021; Sanidad et al. 2022) but also immunopathology (Lim et al. 2021), suggesting that acquisition of tolerance during the critical window has been lost (Lim et al. 2021; Brodin 2022). We therefore also explore what circumstances might select for plasticity in maintaining the critical window open in response to direct pathogen exposure. We discuss our results in the context of observations on immune dysfunction and its determinants and distribution.

## Methods

### THE LIFE CYCLE

To investigate the evolution of critical windows, we develop a discrete time model blending infectious and demographic transitions, extending theory (Klepac and Caswell 2010) previously used to address questions in public health (Metcalf et al. 2012), wildlife diseases (Hobbs et al. 2015), and host immune evolution (Metcalf and Jones 2015). Within the model, individuals are classified by their epidemiological state and their age. There are nine possible epidemiological states: “susceptible” (i.e., not infected by any microbe) denoted *S*, infected by commensal microbes, denoted *M*, infected by the pathogenic microbe, denoted *P*, or infected by both, denoted *MP*. Following infection by any microbe, hosts may enter a state that is temporarily protective against reinfection, *R*. The latter four states may also have acquired tolerance by experiencing infection by any type of microbe during the critical window, indicated by the prefix h (*hM*, *hP*, *hMP*, *hR*; Fig. 1). Note that we model a single commensal microbe as a representative of acquiring sufficient diversity to seed a healthy microbiome, and we model a total of *T* = 25 age classes.

Across each timestep in the model, we track how hosts move between the different states, by becoming infected by either type of microbe (epidemiological transitions; Fig. 1B; Section S1), and by aging (moving up one age class), being born (into the *S* state in the absence of vertical transmission, or into categories *M*, *P*, or *MP* in the presence of vertical transmission), or dying (demographic transitions, Sections S3 and S4). The full population vector of states **n** for a model with *T* age classes consists of 9 × *T* rows, and is given by

(1) $$\begin{array}{ccc} n & = & (S_{1},P_{1},M_{1},MP_{1},R_{1},hP_{1},hM_{1},hMP_{1},hR_{1},S_{2},P_{2},M_{2},\\ & & MP_{2},R_{2},hP_{2},hM_{2},hMP_{2},hR_{2},\;\text{…},\;hM_{T},hMP_{T},hR_{T}), \end{array}$$

where the subscript indicates the age class (see Section S1).

Transitions between the states depend on demographic (Sections S3 and S4) and epidemiological (Section S2) transition probabilities; subscripts reflect different rates according to host infection status (*M* or *P*). Probabilities are independent from host age, except for reproduction—we assume that hosts are iteroparous and start reproducing at age *A*_f_ at rate *f* (Section S4)—and the mortality cost of maintaining the critical window open, *c*, paid only during the window. The latter is added to a baseline mortality μ_b_ and other mortality probabilities governed by different contributions according to infection status. Epidemiological transition probabilities include vertical transmission probabilities (*v*), horizontal transmission rates (β), recovery probabilities (γ), and reinfection scaling (*ϵ*). In the absence of vertical transmission, hosts are born susceptible (denoted *S*), and then may acquire a commensal microbe (thus entering a state denoted *M*) or a pathogenic microbe (entering a state denoted *P*). The probability of microbe acquisition (φ) in each case is defined by prevalence and transmission rates (given by β) of each type of microbe (see Table 1; Fig. 1). For example, the probability that a susceptible individual is infected by the pathogenic microbe φ_p_ is defined by the transmission rate of the pathogenic microbes (β_p_) and the number of individuals that are infected by the pathogenic microbe, where the absence of the age subscript indicates the sum of individuals in each state over all ages:

(2) $$\varphi_{p}=\;1-\exp\left(-\beta_{p}\left(P+hP+0.5\left(MP+hMP\right)\right)\right),$$

assuming for simplicity that dually infected individuals (*MP*, *hMP*) contribute half as much as singly infected individuals. The expression is of the form 1−exp(−r) as we are converting the calculated rate of infection *r* to a probability given the discrete time formulation; see Section S2 for more detail. For simplicity and biological realism, we assume that there is a refractory period during and after infection so that immediate co‐infection does not happen. However, after this period, individuals in state *P* or *h* *P* may be infected by commensal microbes, entering state *MP* or *h* *MP*, according to probability φ_m_, and likewise for individuals in state *M* or *h* *M* . We also assume that infection with any microbe can eventually lead to a state that is (temporarily) protective against re‐infection with either commensal or pathogenic microbes, denoted *R* or *dR*. This framing equates to assuming that the immune system cannot discriminate between microbes (Metcalf and Koskella 2019). In reality, protection against reinfection is likely to be to some degree microbe specific, as will the degree to which hosts acquire tolerance. However, the scenario investigated reflects the situation where selection to balance across the ecology of pathogenic and commensal microbes is the most extreme, and thus the most revealing of the processes at play, as well as reflecting greater tractability. The full set of possible transitions between epidemiological states can be expressed in matrix form (see Section S2).

**Table 1:** Parameter symbols, descriptions, baseline values, ranges explored, and broad impacts, noting that counterintuitive impacts may emerge. Construction of the full model also requires definition of the total number of age classes considered, *T*, the age of fertility, *A*_f_, and the length of the critical window, *W*. The range of parameter values explored is set such that host population extinction is unlikely, and extreme outcomes (a window of length zero, or a window lasting until the onset of reproduction) are encompassed but do not dominate outcomes

Symbol	Description	Baseline and/or Range	Impact of Increasing This Parameter on the Critical Window
μ_b_	Background probability of mortality in one timestep, set to reflect a life expectancy of 100 timesteps in the absence of all other sources of mortality	0.01	Negligible
μ_p_	Mortality probability associated with pathogen infection during the critical window in one timestep, exploring a range from no effect to a maximum reduction in life expectancy of five timesteps	0.005 [0, 0.005]	Reduces the upper age to reduce risk of pathogen infection during the window
μ_p2_	Mortality probability associated with pathogen infection after the critical window in hosts that have acquired tolerance in one timestep, exploring a range from no effect to a maximum reduction in life expectancy of 45 timesteps	0.0075 (modeled as 1.5 × μ_p_)	Increases or reduces the upper age
μ_m_	Mortality probability associated with microbe infection after the critical window in hosts that have not acquired tolerance in one timestep, exploring a range from no effect to a maximum reduction in life expectancy of five timesteps	0.005 [0, 0.005]	Increases the upper age to increase opportunity for acquiring tolerance via microbe or pathogen infection during the window
c	Mortality probability reflecting the cost associated with keeping the window open in one timestep, assumed to be of the same scale as the cost of infection during the window	0.0005	Reduces the upper age to reduce the mortality cost
β_p_	Magnitude of transmission of the pathogen (a rate per timestep, converted to a probability, see main text) exploring a range from 1.2 to 3.5 individuals infected by one infectious individual in a completely susceptible population, from low transmission to transmission commensurate with pathogens like influenza	1.5 [1.2, 3.5]	Allows reduction of the upper age (thus reducing costs of keeping the window open) by reducing the age of first pathogen infection
β_m_	Magnitude of transmission of the neutral microbe (a rate per timestep, converted to a probability, see main text); as above	1.5 [1.2, 3.5]	Allows reduction of the upper age (thus reducing costs of keeping the window open) by reducing the age of first microbe infection
*ϵ*_p_	Parameter adjusting the probability of reinfection for the pathogen; the full potential range if explored	0.95 [0, 1]	Reduces the average age of first infection so predominantly a reduction
*ϵ*_m_	Parameter adjusting the probability of reinfection for the neutral microbe; the full potential range if explored	0.95 [0, 1]	Reduces the average age of first infection but also increases the probability of repeat infections of hosts that have not acquired tolerance, so predominantly increases the upper age
γ_p_	Probability of recovery for the pathogen in one timestep; the full potential range if explored	0.95 [0, 1]	Context dependent as can both reduce the average age of first infection and increase the probability of reinfection
γ_m_	Probability of recovery for the neutral microbe in one timestep; the full potential range if explored	0.95 [0, 1]	Context dependent as can both reduce the average age of first infection and increase the probability of reinfection
*v*_p_	Probability of vertical transmission of the pathogenic microbe from an infected mother^1^; the full potential range if explored	[0, 1]	Acts similarly to the magnitude of transmission
*v*_m_	Probability of vertical transmission of the commensal microbe from an infected mother^1^; the full potential range if explored	0.95 [0, 1]	Acts similarly to the magnitude of transmission
*f*	Fertility rate per timestep, exploring a range from species that just achieves replacement over its life span, to one with very high fertility	2 [0.12, 2]	Shifts the average age down
*a*_g_	Aging probability per timestep	1	
α	Magnitude of seasonal forcing, exploring a range from no seasonal forcing to very strong annual swings, at the extremes of what is reported for childhood infections	0 [0, 0.7]	Increases the average age of infection as individuals will age through periods of low transmission

^1^ See the Supporting Information for discussion of approach to dual infections.

Acquisition of microbes may be associated with shifts in the host's mortality hazard in three ways (indicated by lightning bolts in Fig. 1A) that will directly shape the optimal critical window length (i.e., age at which the critical window should end), denoted *W*. Pathogens acquired during the critical window may be associated with higher mortality, as the host's defenses are diminished during the process of acquiring tolerance (Fig. 1A, i); if they are re‐acquired subsequent to infection by either commensal microbes or pathogen, this will result in higher risks of mortality, as hosts that have acquired tolerance will not rally adequate defenses (Fig. 1A, iii). Commensal microbes not seen during the critical windows and acquired later will be associated with higher mortality risks as a result of not having acquired tolerance, and thus will drive immunopathology (Fig. 1A, ii). Other life cycle parameters will also shape the optimal length as a result of distribution of the risk of infection across age.

The combination of all these demographic and epidemiological transition probabilities determines transitions between the states. As an illustration, the number of susceptible individuals in the first age class after one timestep (and assuming that the first age class is within the critical window) reflects a combination of demographic and epidemiological transitions:

(3) $$\begin{array}{ccc} S_{1\left(t+1\right)} & = & S_{1\left(t\right)}\;\left(1-a_{g}\right)\left(1-\varphi_{p}\right)\left(1-\varphi_{m}\right)\left(1-\mu_{b}-c\right)\\ & & +f\sum_{A_{f}}^{T}n_{a}. \end{array}$$

In equation (3), the first term captures 1‐year‐old susceptible individuals at time *t*, $S_{1(t)}$, not aging ($1-a_{g}$), not being infected by either pathogenic or commensal microbes $\left(1-\varphi_{p}\right)\left(1-\varphi_{m}\right)$, and not dying either as a result of the background probability of mortality or the probability associated with the cost of being in the critical window ($1-\mu_{b}-c$) noting that this expression is constrained to be greater than zero. The second term captures birth of new susceptible individuals from all individuals that have reached the age of maturity, *A*_f_ (assuming no vertical transmission occurs). Moving from the equation to the matrix framing is described in Section S3. Similarly, *P*_1_ after one timestep is given by

(4) $$\begin{array}{ccc} \;P_{1\left(t+1\right)} & = & P_{1\left(t\right)}\;\left(1-a_{g}\right)\left(1-\gamma_{p}\right)\left(1-\mu_{p}-\mu_{b}-c\right)\\ & & +\;S_{1\left(t\right)}\left(1-a_{g}\right)\varphi_{p}\left(1-\varphi_{m}\right)\left(1-\mu_{b}-c\right)\\ & & +\;MP_{1\left(t\right)}\left(1-a_{g}\right)\gamma_{m}\left(1-\gamma_{p}\right)\left(1-\mu_{b}-c\right)\\ & & +\;R_{1\left(t\right)}\left(1-a_{g}\right)\in_{p}\varphi_{p}\left(1-\in_{m}\varphi_{m}\right)\left(1-\mu_{b}-c\right).\;\; \end{array}$$

In equation (4), the first term captures individuals in the first age class infected with the pathogen at time *t*, $P_{1(t)}$, not aging ($1-a_{g}$), not recovering ($1-\gamma_{p}$), and not dying ($1-\mu_{b}-c-\mu_{p}$) noting that this last expression is constrained to be greater than zero; the second term reflects susceptible individuals at time *t* in the first age class, $S_{1(t)}$, not aging ($1-a_{g}$), becoming infected with a pathogenic microbe (ϕ_p_), not becoming infected with a commensal microbe ($1-\varphi_{m}$), and not dying ($1-\mu_{b}-c$); the third term reflects doubly infected individuals $MP_{1(t)}$ recovering from infection by the commensal microbe but not the pathogenic microbe, $\gamma_{m}\left(1-\gamma_{p}\right)$, and not dying and not aging as in the previous; the fourth term captures recovered individuals, $R_{1(t)}$ being (re)infected by the pathogen *ϵ*_p_ *φ*_p_, and not being (re)infected by the commensal (1–*ϵ*_m_ *φ*_m_), and not aging or dying as in the previous. Again, details on transition in the matrix form are provided in Section S3.

Similar logic is used to define transitions for all other states. The full set of epidemiological transitions associated with survival and aging can be conveniently expressed using a matrix population model framing (see Sections S2 and S3) and the same can be achieved for reproduction (see Section S4).

### IDENTIFYING THE EVOLUTIONARILY OPTIMAL LENGTH OF THE CRITICAL WINDOW (W*)

To estimate fitness associated with a particular value of the length of the critical window *W*, we first initialize the population vector n with one individual in each state, and then iterate this population out to equilibrium by matrix multiplication (see Section S5). The equilibrium is defined as occurring when the change in the one timestep growth rate falls below 10^−9^. We then calculate the population rate of increase λ, or the dominant eigenvalue of the population transition matrix, which provides an estimate of fitness (Klepac and Caswell 2010). For each scenario considered (e.g., combination of host demography, pathogenic and commensal microbe transmission, etc.), we estimate fitness associated with the full range of possible lengths of the window *W*, from there being no window (*W* = 0) to the window being the size of the largest age class modeled (*W* = *T*) in steps corresponding to the age class bins used. With this fitness profile in hand, we locate the length of the window *W* with the highest long‐term fitness as measured by λ. This is referred to as the optimal window length, W*.

### PARAMETERS EXPLORED

We ground our analysis in a life history where the baseline probability of mortality in the absence of other mortality risks (from pathogen, commensal microbe, and cost of maintaining the window) is $\mu_{b}=\;0.01$ (corresponding to a life expectancy of around 100 timesteps), and with fecundity starting at $A_{f}=20$ timesteps and producing f=2 offspring per individual and maximum age class of T=25. We allow mortality associated with the pathogen and commensal microbes to attain a maximum value of 0.005, that is, equivalent to constant exposure to each hazard reducing life expectancy by around a third. We set the cost of maintaining the window open to c=0.0005, that is, a tenth of this quantity. Within these bounds, we explore a wide range of parameter space (see Fig. S1; Table 1)

Optimal window length W* is directly affected by the cost of keeping the window open (associated with a mortality hazard *c*), and the mortality costs of different outcomes: (i) mortality for hosts infected by the pathogen during the critical window (μ_p_); (ii) mortality for hosts that have not acquired tolerance infected by the commensal microbes after the critical window (μ_m_); and (iii) mortality for hosts that have acquired tolerance infected by the pathogen after the window (μ_p2_) (see Fig. 1A,B, indexed correspondingly). The magnitudes of transmission of both the commensal microbes (β_m_) and the pathogen (β_p_) also affect the optimal window length as they affect the average age of infection and concentration of risk over age. This is also the case for the prospects of vertical transmission from infected adults to offspring for the pathogen (*v*_p_) and commensal microbes (*v*_m_). Similarly, the probabilities of recovery (γ_p_ and γ_m_) and the risk of being reinfected following prior infection (*ϵ*_p_ and *ϵ*_m_) will shape the optimal length of the window by altering the profile and concentration of risk over age. All of these effects will be modulated by the impacts of demography within the population, in particular fertility, *f*, by driving the supply of new susceptible individuals. We also explore the impact of seasonal fluctuations in transmission α (Section S6). For all parameter values and ranges explored, see Table 1.

### EVALUATING THE FITNESS CONSEQUENCES OF PLASTICITY IN CRITICAL WINDOW MAINTENANCE

We also evaluate the circumstances where plasticity in maintenance of a critical window dependent on the microbial environment experienced early in life increases fitness. We explore the scenario where infection of an individual by the pathogen before the end of the critical window can cause the critical window to close. We assume that such plastically responsive individuals avoid excess pathogen‐associated mortality usually incurred during the critical window, as well as the costs of maintaining the window open, but pay the costs of not acquiring tolerance. This alteration requires modifying the matrix model framework (Section S7) so that transitions out of the susceptible class (indicating a first infection) associated with pathogen infection result in a different mortality background. This can be achieved by introducing four extra epidemiological classes that individuals may enter into as a result of a plastic response (see Section S6). With this extended model in hand, we estimate fitness across the range of lengths of the critical window for plastic and nonplastic strategies.

## Results

### OPTIMAL STATIC WINDOW

We start by focusing on the direct effects of the different sources of mortality on optimal length of the critical window W*. We frame our comparisons relative to a baseline scenario and associated optimum (Fig. 1C, black line; see Table 1 for parameters). Doubling pathogen‐associated mortality in hosts during the critical window (μ_p_) reduces the optimal upper age of the critical window W* to reduce the time during which individuals are at risk of dangerous pathogen infection (Fig. 1C, red line, indexed [i]); doubling mortality of hosts that have not acquired tolerance infected by the commensal microbes increases the upper age of the critical window to increase the opportunity for acquiring tolerance before the end of the window (Fig. 1C, purple line, indexed [ii]); and doubling mortality of hosts that have acquired tolerance infected by the pathogen reduces the window to reduce occurrence of acquisition of tolerance (Fig. 1C, red line, indexed [iii]). Results across a wide range of values of mortality are shown in Figure S1.

**Figure 2:**
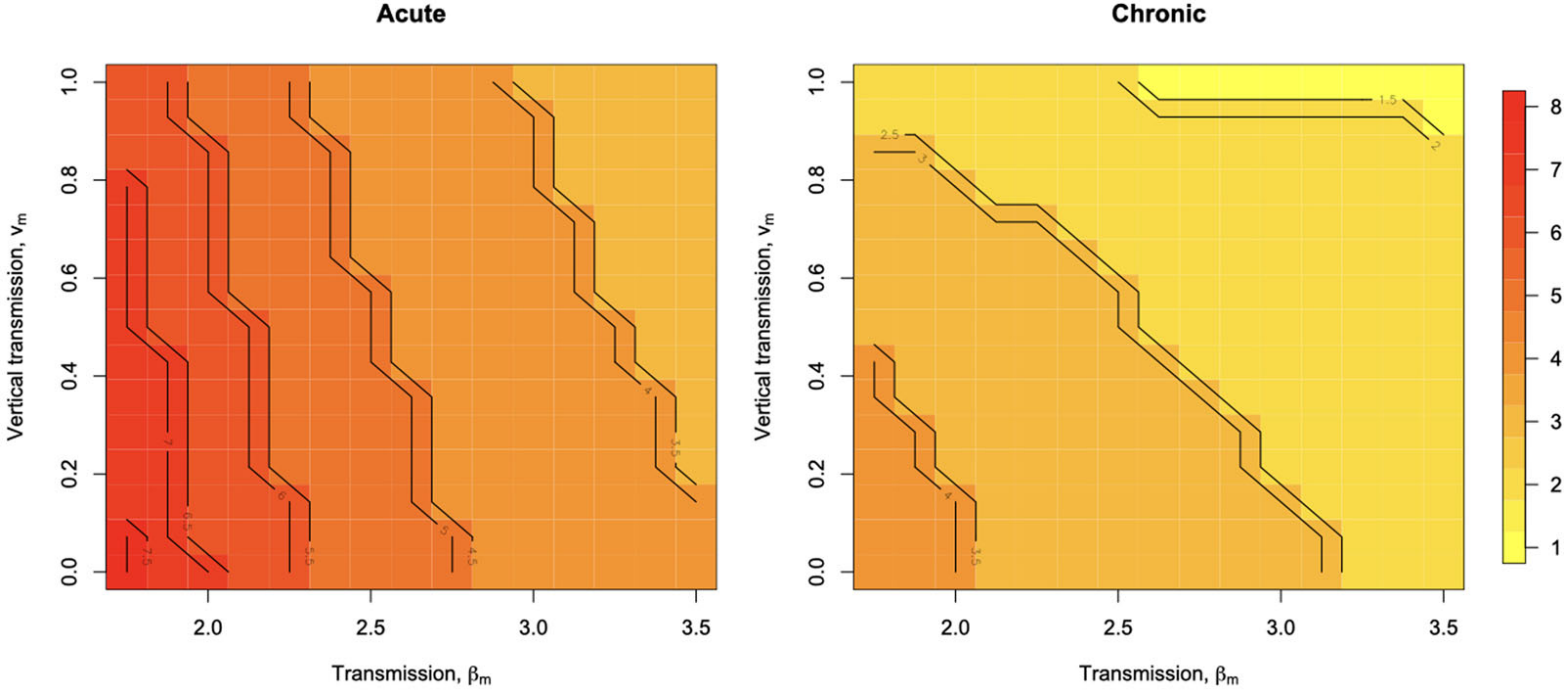
Commensal microbial ecology effects on optimal length of the critical window W* in the absence of the pathogen across increasing transmission (β_m_, horizontal axis) and increasing vertical transmission (*v*_m_, vertical axis) comparing a scenario with acute commensal microbe infection ($\gamma_{m}=0.95$, left) and chronic commensal microbe infection ($\gamma_{m}=0.05$, right); all other parameters set to the baseline shown in Table 1, except that $\beta_{p}=0$. In both cases, the optimal length of the critical window W* declines with increases in transmission (horizontal axis) and vertical transmission (vertical axis) as this increases the probability that hosts acquire tolerance young (colors represent optimal window length W*, with the yellow to red gradient indicating a transition from low to high ages; contours and legend show values). Chronic commensal infections support even younger optimal ages W*.

Next, we consider the indirect impacts of microbial ecology. For clarity, we first focus on the case where only commensal microbes are present. Increases in both vertical transmission *v*_m_ and horizontal transmission β_m_ reduce the average age of infection, the former directly, and the latter by increasing the rate at which individuals are infected. These effects increase the probability that individuals acquire commensal microbes earlier in life, thus reducing the degree to which hosts suffer later life mortality associated with infection in individuals that have not acquired tolerance, μ_m_. Increases in both forms of transmission thus have the effect of reducing the optimal upper age of the window W* (Fig. 2), as this has the benefit of reducing the amount of time that the cost *c* of maintaining the window open is paid (see Table 1). The pattern is observed for both acute commensal microbe infections (where clearance of infection is rapid, $\gamma_{m}=0.95$; Fig. 2A) and chronic commensal microbe infections (where once an infection is acquired, it tends to be kept for life, $\gamma_{m}=0.05$; Fig. 2B). However, the latter sets the optimal length of the window W* to a lower level than the former (Fig. 2A vs. 2B), because the overall effect of reduced clearance is an increase in prevalence, and thus an increase in the probability that a susceptible individual will be infected early in life.

**Figure 3:**
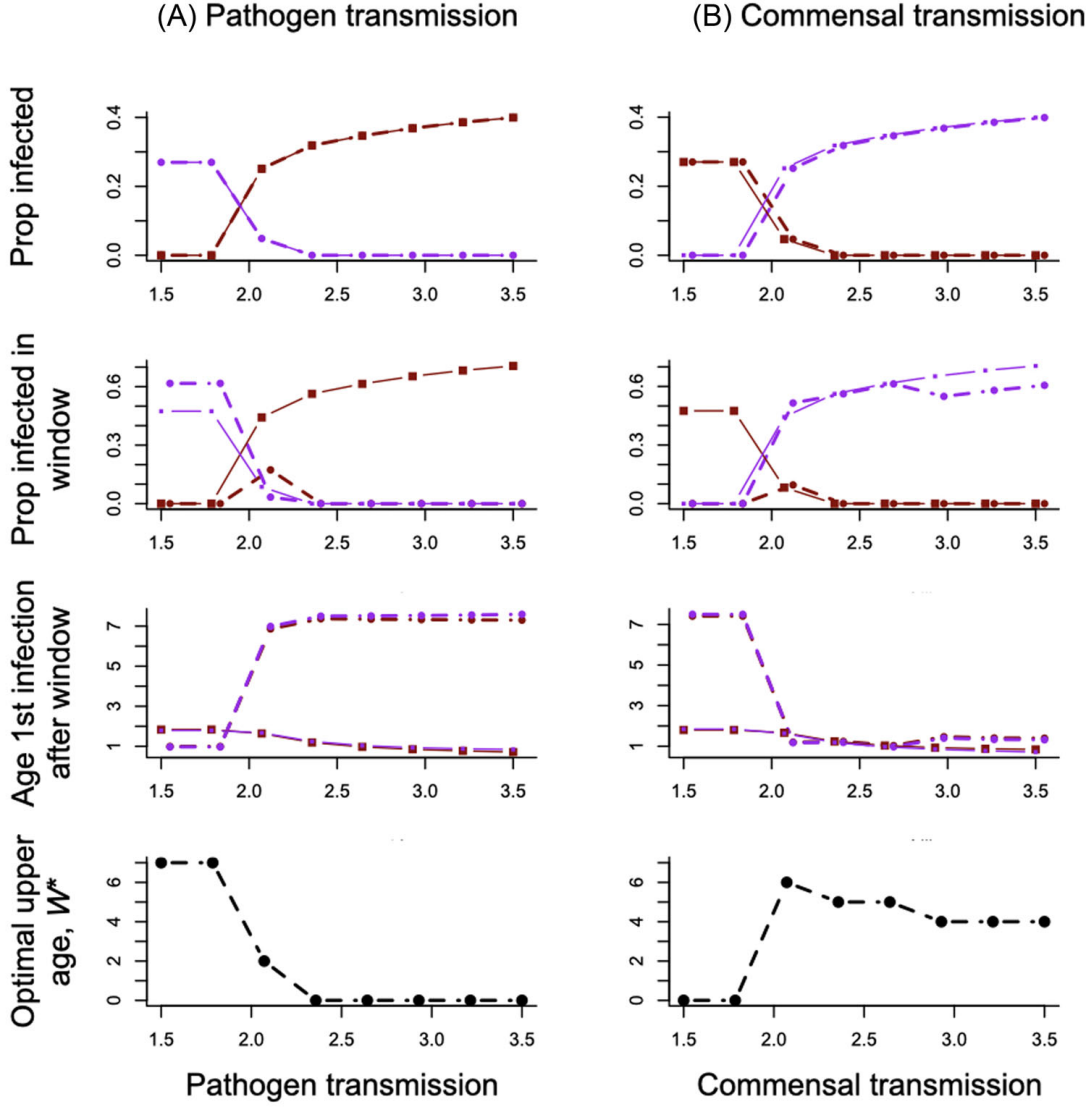
Indirect impacts of altering transmission of (A) the pathogen and (B) the commensal microbes with magnitude of transmission shown on the horizontal axis. All other parameters are set to the baseline in Table 1, except that $\beta_{m}=\beta_{p}=2$. Solid lines and squares indicate outcomes for *W* = 6; dashed lines and circles indicate outcomes for *W* set to the optimum for each parameter combination, with red reflecting the pathogen, and purple the commensal microbe; this optimal age W* is plotted in the last row. Points are slightly jittered, and of different sizes for visibility. Increasing transmission increases the proportion infected by the pathogen (/commensal microbe) and has the concomitant effect of reducing the other microbe (first row, solid and dashed red lines increase in A and decrease in B, and vice versa for the purple lines). For *W* = 6, increasing transmission also increases the proportion infected in the critical window (second row, solid lines, A and B), but at the optimum W*, different outcomes occur (dashed lines) with more infection when commensals dominate host mortality risk, and less when pathogens dominate. Although increasing transmission drives a reduction in the first age of infection after the window if the window age is unchanged at *W* = 6, simply because microbes will be acquired faster (solid lines, A and B), setting *W* to the optimum (dashed lines) can increase or reduce this age again depending on whether pathogen or commensals dominate host mortality risk. The final row shows the optimal age W* declining as pathogen transmission increases to minimize pathogen infection in the window or in hosts that have acquired tolerance, and first increasing then decreasing with increases in commensal microbe transmission to balance the costs of maintaining the window open given impact on the ecology of the pathogen and commensal microbe.

Next, we consider changes in features of microbial ecology when both the commensal microbes and a pathogen are present. To reveal the underlying drivers of the optimal window length W*, we depict changes in the total proportion infected (Fig. 3, top row), and proportion infected in the window (Fig. 3, second row), and changes in the age at which the hosts that have not acquired tolerance are infected (Fig. 3, third row). We estimate these quantities both at an identical window length (set to *W* = 6) and at the optimal age for each parameter combination (dashed lines on Fig. 3). This optimal age W* is shown in the last row of Figure 3.

Increased transmission of either the pathogen (horizontal axis of the first column, β_p_) or the commensal microbes (horizontal axis, second column, β_m_) increases the proportion of individuals infected with the focal microbe (Fig. 3A,B, first row). The transient cross‐protection assumed (see *Methods*) also means that an increase in the pathogen drives a reduction in commensals, and vice versa (red and purple lines intersect when the horizontal axis corresponds to transmission of the nonfocal microbe, here set to β=2). If the window is unchanged, increases in both forms of transmission drive a reduction in the average age of infection, including in the age of the first infection after the window (Fig. 3, third row, solid lines). The effect of shifting the window length to the optimum for each parameter combination is a shift in the distribution of infection across age (dashed lines, rows 1–3, Fig. 3)

Increasing pathogen transmission drives a decline in W* (Fig. 3, bottom left) driven by increasing incidence and further enabled by declining average age of infection with the pathogen. When commensal microbe transmission is so low as to make the probability of commensal infection negligible, the optimal window W* is very short, and then increases sharply at the threshold when commensal transmission is sufficient to mean that the risk of infection in hosts that have not acquired tolerance must be reduced (Fig. 3, bottom right). The optimal window W* then starts to decline as increases in transmission result in a lower average age of infection of the commensal microbes permitting a shorter critical window because hosts acquire tolerance early in life (Fig. 3, bottom right), meaning that a smaller cost of maintaining the window open needs to be paid (i.e., the mortality hazard *c*; see Table 1). Vertical transmission has broadly similar effects (not shown). Results across a wide range of values of transmission are shown in Figure S1. Probabilities of recovery and waning have context‐dependent effects as a result of their effects on both age and prevalence, also shown in Figures S1 and S2.

**Figure 4:**
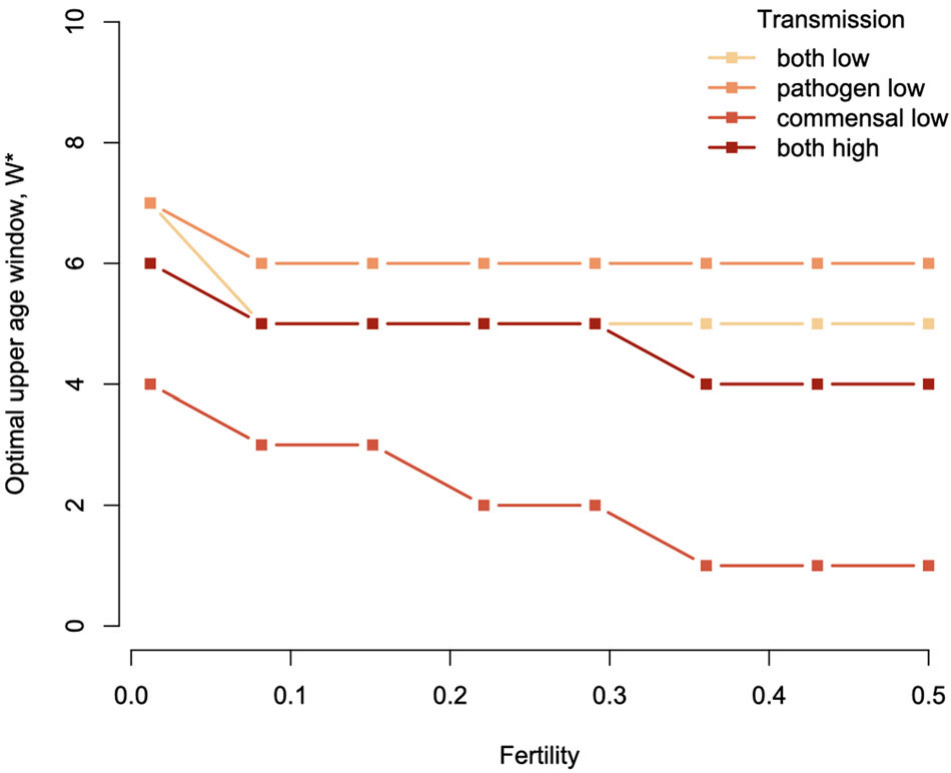
Effects of host fertility on optimal length of the critical window. As fertility *f* increases (horizontal axis), the average age of infection declines, driving a decline in optimal length of the critical window W*. The rate of decline hinges on the relative abundance of pathogen and microbe, which in turn hinge on their magnitude of transmission (colors, legend); with low transmission corresponding to β=2 and high transmission corresponding to β=2.1, all other parameters set to the baseline in Table 1. Predominance of commensal microbes increases optimal window length, and predominance of pathogens reduces it.

Host demography will also shape the optimal window length W* via its effects on pathogen incidence and distribution across age. Increasing host fertility (birth rate) drives down the average age of infection, enabling a decline in the optimal length of the susceptible window (Fig. 4). The magnitude and pattern of decline depend on the prevalence of the commensal microbe and pathogen.

**Figure 5:**
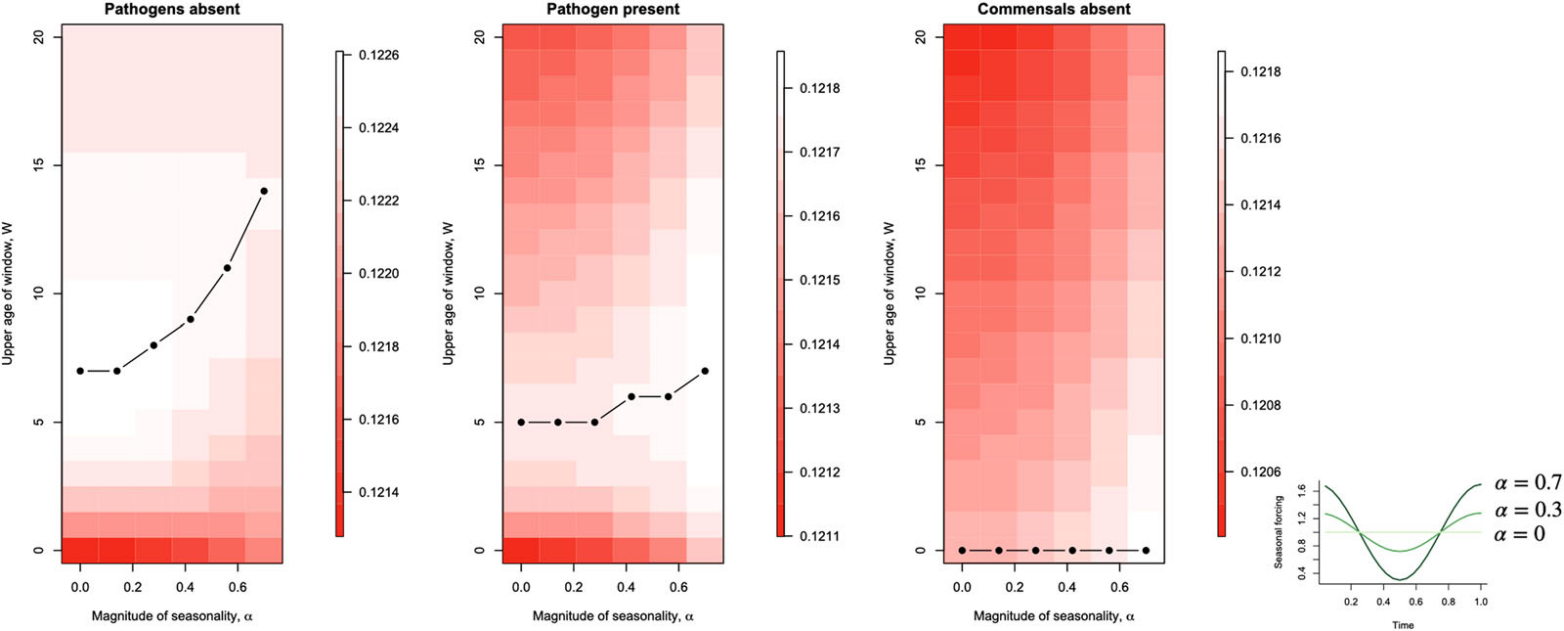
Effects of seasonal fluctuations in transmission on optimal length of the critical window. As the magnitude of seasonal variation in transmission increases (horizontal axis on each plot; with the multiplier of transmission over the course of a year illustrated by the inset on the right), the optimal length of the critical window W* increases if only commensal microbes are present (left hand plot, vertical axis indicates the length of the critical window, *W*, and whiter colors correspond to higher fitness; the optimum is shown as the solid line), with $\beta_{m}=2$ and $\beta_{p}=0$, all other parameters set to the baseline in Table 1. This occurs because seasonality drives troughs in incidence during which some individuals may not acquire tolerance. If pathogens are also present ($\beta_{m}=2$ and $\beta_{p}=2$, all other parameters as before), this increase in W* is less marked, and the optimum in the absence of the commensal microbe ($\beta_{m}=0$ and $\beta_{p}=2$) is that the window collapses. Baseline parameters reflect an acute commensal and pathogen infection ($\gamma_{m}=\gamma_{p}=0.95$), which is expected to result in the largest impact as most compressing duration of infection.

Finally, we consider the role of seasonal fluctuations in transmission. When pathogens are absent or rare, large fluctuations in transmission over the course of a year extend the optimal length of the window W* to ensure exposure to commensals for most hosts before the end of the critical window despite periods of the year when prevalence is low (Fig. 5, first column). Including pathogens diminishes this extension of the optimal window length due to the countervailing selection pressure to reduce window length (Fig. 5, second column). Finally, if there are no commensals, the optimum is to reduce the window to nothing (Fig. 5, last column).

### CONTEXTS THAT FAVOR PLASTICITY IN ENDING THE CRITICAL WINDOW

**Figure 6:**
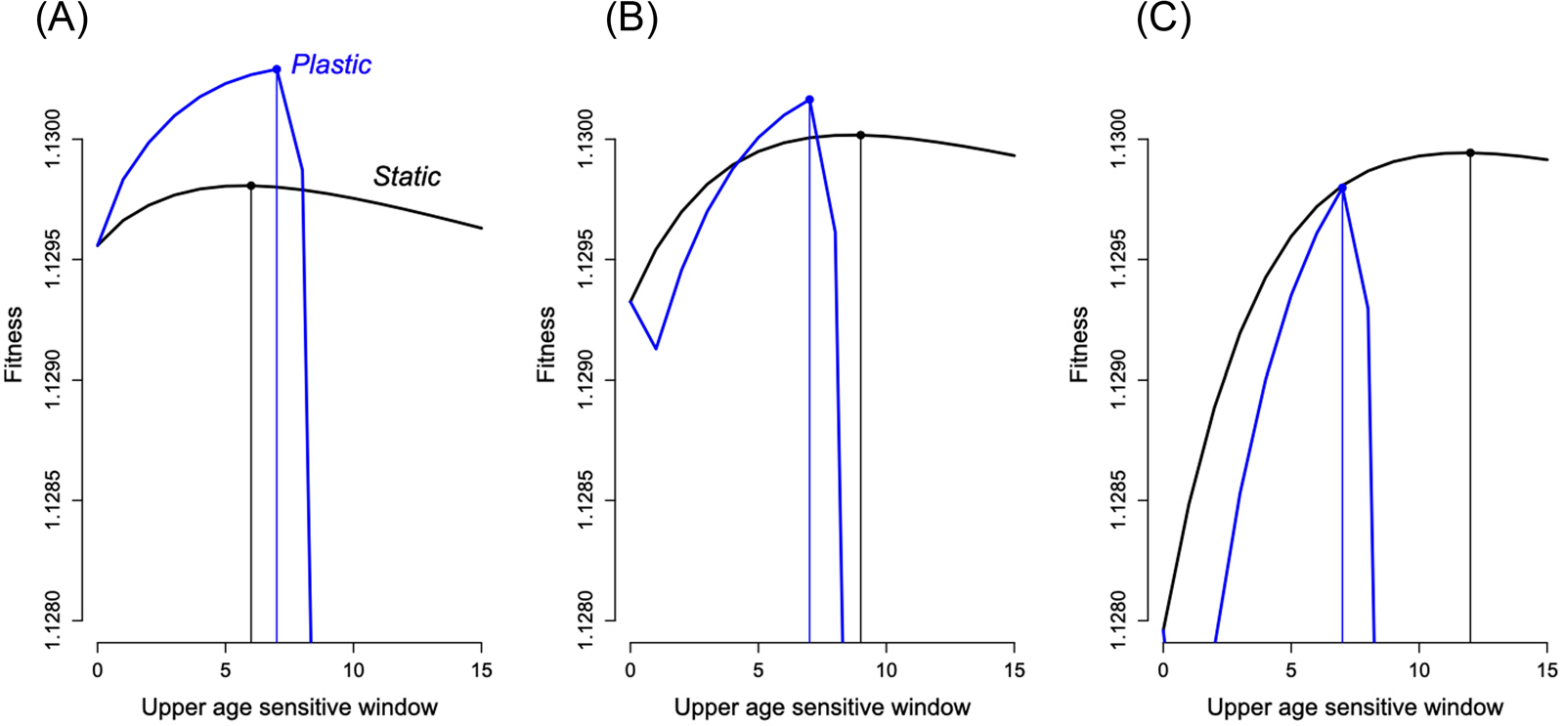
Fitness (vertical axis) of plastic (blue) and nonplastic (black) strategies across a range of lengths of the critical window (horizontal axis) for (A) a baseline (parameters shown in Table 1, and baseline in Fig. 1C); (B) the same but with mortality associated with immunopathology (μ_m_) doubled and mortality associated with pathogen associated mortality (μ_p_ and μ_p2_) halved; (C) the same but with mortality associated with immunopathology (μ_m_) multiplied by five and mortality associated with pathogen associated mortality (μ_p_ and μ_p2_) divided by a third. The spectrum of results indicates that the optimal length of the window for plastic strategies may be shorter or longer than the nonplastic strategy, and if the costs of immunopathology are sufficiently high, and the costs of pathogen‐associated infection are sufficiently low, the plastic strategy can have lower fitness than the nonplastic strategy.

Contrasting the fitness profile of life cycles with plasticity (where first infection with the pathogen that occurs during the critical window can close the critical window) with those without plasticity reveals generally higher fitness of plastic responses, especially where the length of the critical window can change (Fig. 6A, blue lines generally higher than black lines). However, the advantage of the plastic response is diminished under conditions of higher immunopathology associated with infection in hosts that have not acquired tolerance (μ_m_) and diminished mortality associated with pathogen infection (μ_p_ and μ_p2_). These may be sufficiently high as the result in consistently lower fitness of the plastic strategy relative to the nonplastic strategy (Fig. 6C, the blue line is lower than the black line across all lengths of the critical window).

## Discussion

Animal models indicate the existence of a narrow window early in life during which exposure to microbes results in acquisition of tolerance. Here, we asked what selection pressures define how long this critical window should evolve to be. We demonstrate that the optimal length hinges on the direct costs of infection with pathogen and commensal species both during the critical window and afterward. We also reveal the importance of features of microbial ecology, and illustrate contexts that might favor selection toward plasticity in maintaining a critical window.

A number of predictions emerge for our mathematical model framing. First, our analysis aligns with predictions that declining abundance or diversity of commensals could be contributing to population scale increases in immune dysfunction (Blaser and Falkow 2009; McDade 2012), as also suggested by specific empirical case studies. For example, children with “immature” microbiomes relative to their age (Stokholm et al. 2018) or who have been exposed to antibiotics (Donovan et al. 2020; Patrick et al. 2020), both likely to translate into reduced exposure to commensals, have increased risk for asthma. Over evolutionary time frames, if the environment experienced by an individual's descendants does not resemble the context in which selection occurred as a result of “disappearing microbes” (Blaser and Falkow 2009), then the evolved window length will be too short, and late life immune dysfunction is expected, a phenomenon sometimes termed “evolutionary mismatch.” Evolutionary mismatch may, in some contexts, be mitigated by plasticity. Although there is some empirical evidence suggestive of plasticity in how tolerance is acquired in response to pathogen infection *i* *n utero* (Lim et al. 2021), and we do find that such plasticity would be beneficial in contexts with high risks of pathogen associated mortality (Fig. 6), it is less obvious how plasticity in response to an absence of commensals could operate, or be protective.

Second, taking a comparative framework, we predict that, all else equal, species that evolved in contexts where commensals were rarer, transmission was highly seasonal, and/or fertility was low will have longer critical windows. When vertical transmission of commensals is included in the model (and thus commensals are more likely to be acquired early and consistently), we find the critical window to be reduced. Although evidence on length of critical windows remains rare across species, there may be approaches where one might leverage growing data on the saturation of microbial communities across age. Our predictions regarding the length of critical windows across systems could be tested in terms of microbiome maturation, where longer windows correspond to longer periods of microbiome successional dynamics or variability. Such data have been used, for example, to put forward a 100‐day critical window in humans (Stiemsma and Turvey 2017).

Finally, specific mechanisms involved in defining the end of the window are increasingly well‐described, and may be associated with different mortality costs. For example, MAIT cells recruit in response to a very generalized signal of microbes, that is, production of microbial‐derived intermediates of vitamin B2 (riboflavin) synthesis (Constantinides et al. 2019). This might be less likely to drive an increase of late life pathogen mortality associated with inappropriate acquisition of tolerance to pathogens (excess mortality indexed as [iii] in Fig. 1) than the development of tolerance to specific pathogen antigens that might be much harder for the immune system to counteract. Thus, one might be able to predict differences associated with the predominance of different mechanisms of acquisition of tolerance. Mechanisms will also be differentially amenable to plasticity, and thus might predominate differentially across contexts predicted across Figure 6.

Both the static and plastic models make a number of simplifications. In particular, we ignore the role of symbionts as enablers of immune maturation (Gerardo et al. 2020). Interactions of the microbiome have been described as critical in immune ontogeny in general (Gerardo et al. 2020), and specifically with Th17, for example, described as leading to a state of “controlled inflammation” (Lee and Mazmanian 2010). However, in the scenario where any infection during the sensitive window improves protection against pathogens later in life, the length of the window will be constrained by the cost of keeping the window open against these benefits. We do not engage with, but certainly acknowledge, the potential role of early life exposure to allergens in shaping these processes. For example, it has been suggested that birth during high pollen or dust mite season might drive development of allergies late in life (Vovolis et al. 1999; Yoo et al. 2005). There might be interesting interactions between microbe and allergen seasonality. However, in an analysis of birth seasonality, no signature of risk of asthma was detected (Wjst et al. 2005), possibly a result of the conflicting footprints of the seasonality of microbes and allergens, further complicated by the potential role of seasonal pathogens like Respiratory Syncytial Virus in shaping risk of asthma (Driscoll et al. 2020). We also neglect the role of maternal protection via transfer of antibodies during the early phases of offspring life (transplacentally or during lactation in mammals, or via the egg yolk in birds [Boulinier and Staszewski 2008]). An absence of commensal microbes typically transferred during lactation, such as the *Bifidobacteria*, can lead to systemic inflammation and immune dysregulation early in life (Henrick et al. 2021), which might also translate into late life immune dysfunction. Beyond directly providing commensal bacteria, thus enabling acquisition of tolerance, direct provisioning of offspring via lactation might also reduce opportunities for pathogen introduction. If maternal immunity simply prevents infection very generally (e.g., Zheng et al. 2020), the optimal length of the critical window will be longer, as the risks of pathogen infection are diminished, possibly modulated by the impacts of maternal immunity on transmission (McKee et al. 2015). If maternal immunity allows infection but protects offspring from the effects of microbes in ways that still allow “learning,” then the critical window can be commensurate with the duration of maternal immunity and risks will be minimized. We also ignore all direct costs of plasticity, which might reduce the range of contexts where the plastic strategy was more flexible than the static strategy. Finally, we consider that the immune system cannot distinguish between pathogenic and commensal microbes (Metcalf and Koskella 2019). Another possibility is that pathogenic infection generates no tolerance to future infections, whereas commensal infection does, in which case pathogen infection will only select for reduced window length and commensal infection for greater window length. Situations between these two extremes may generate nuanced intermediate outcomes and more realistic framings are an interesting direction for future study.

To conclude, critical windows are described across the biological sciences, from neuroscience to immunology. They are defined as a time period or life stage where a specific experience shapes a trait to a larger extent than the same experience in other time periods. Models of the evolution of critical windows in neuroscience underscore that they allow individuals to tune their ontogeny to local environmental conditions (Frankenhuis and Walasek 2020). This raises a general point: broad requirements for the evolution of critical windows are that the environment early in life provides useful information about conditions later in life. Knowledge of microbial and host ecology provides a means to predict the expected length of critical immune windows across host species, whereas current changes in the microbial environment—especially general loss of microbial diversity and/or increased risk of pathogen exposure—have the potential to disrupt this predictability (Blaser and Falkow 2009), with potential amplifying effects for human health. A deeper understanding of the features of the evolutionary ecology of critical windows may enable better prediction of the future burden of immune dysfunction.

## CONFLICT OF INTEREST

The authors declare no conflict of interest.

## AUTHOR CONTRIBUTIONS

CJEM, BT, and BK conceived the study. CJEM and MB developed the model. CJEM and BT drafted the initial version of the manuscript. All authors contributed to later versions of the manuscript.

## Supporting information

Supplementary figure S1a

Supplementary figure S1b

Supplementary figure S1c

Supplementary figure S1d

Figure S1: The range of optimum windows W^*^
