# Supplementary figures and images for: Hosts, microbiomes, and the evolution of critical windows
Source: Evol Lett. 2022 Oct 27;6(6):412–25. doi: 10.1002/evl3.298 (PMC9783423; doi:10.1002/evl3.298)

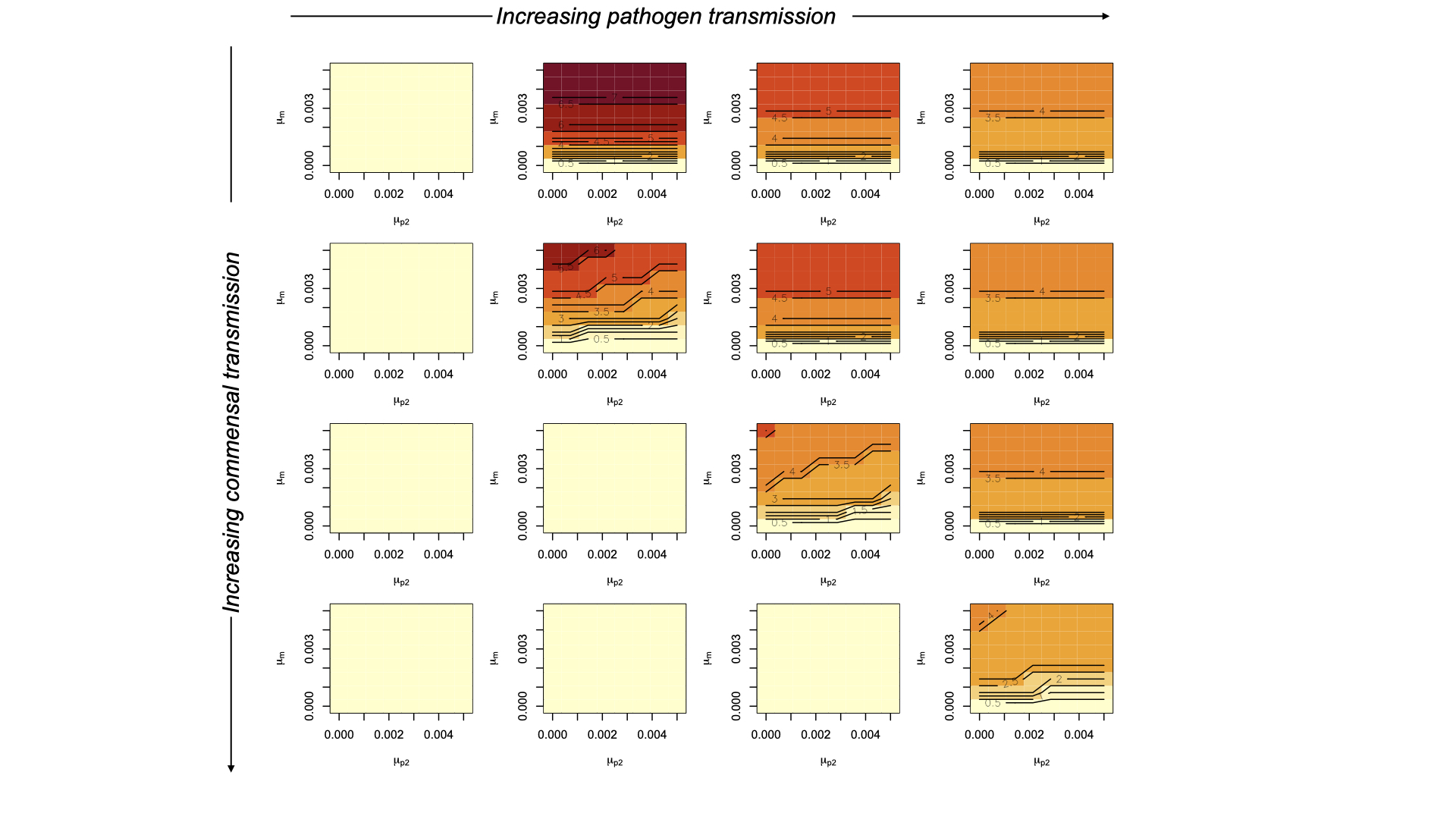

Supplement: Supplementary file 1 — Supplementary figure S1a [file EVL3-6-412-s004.jpeg]

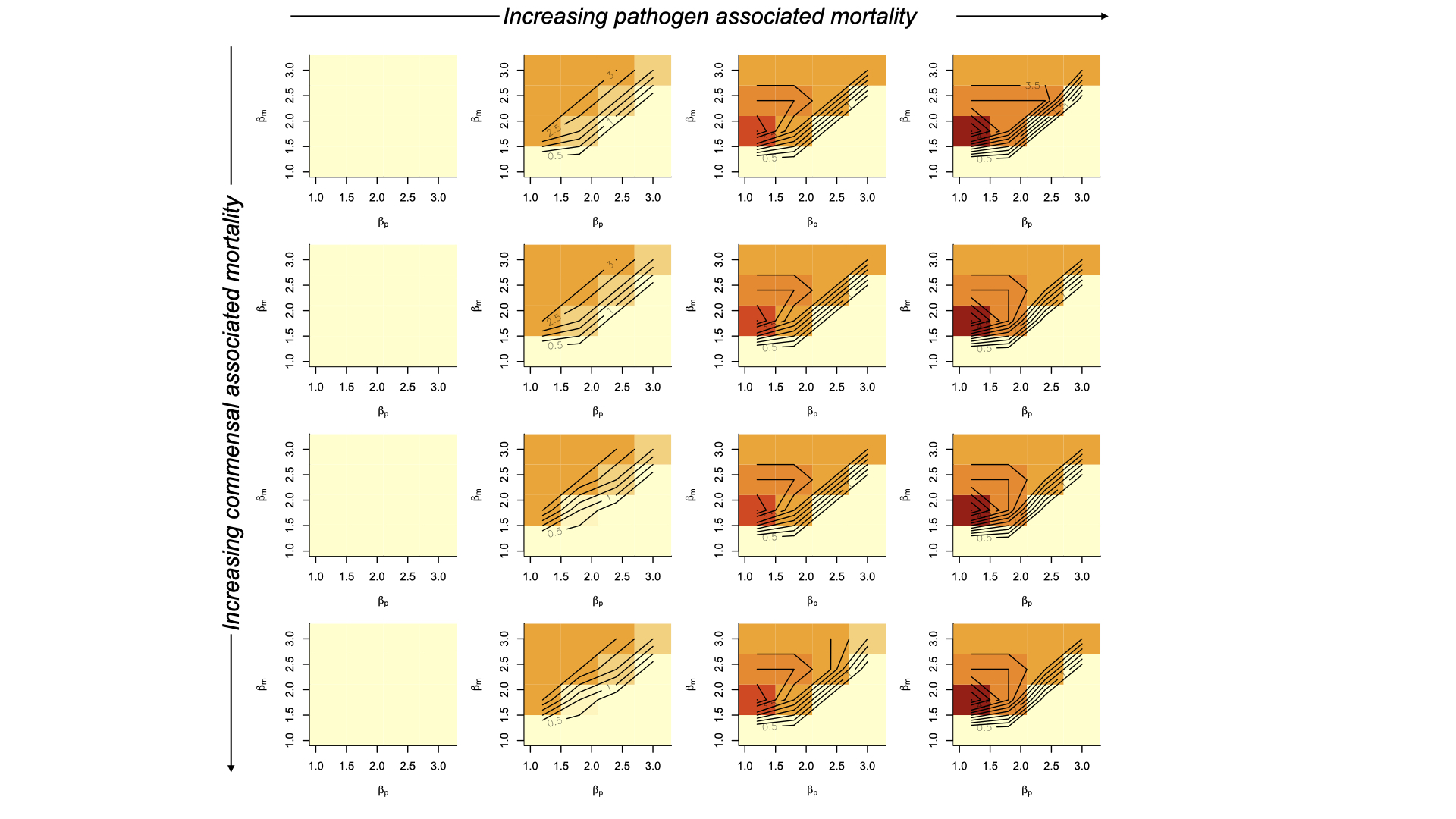

Supplement: Supplementary file 2 — Supplementary figure S1b [file EVL3-6-412-s001.jpeg]

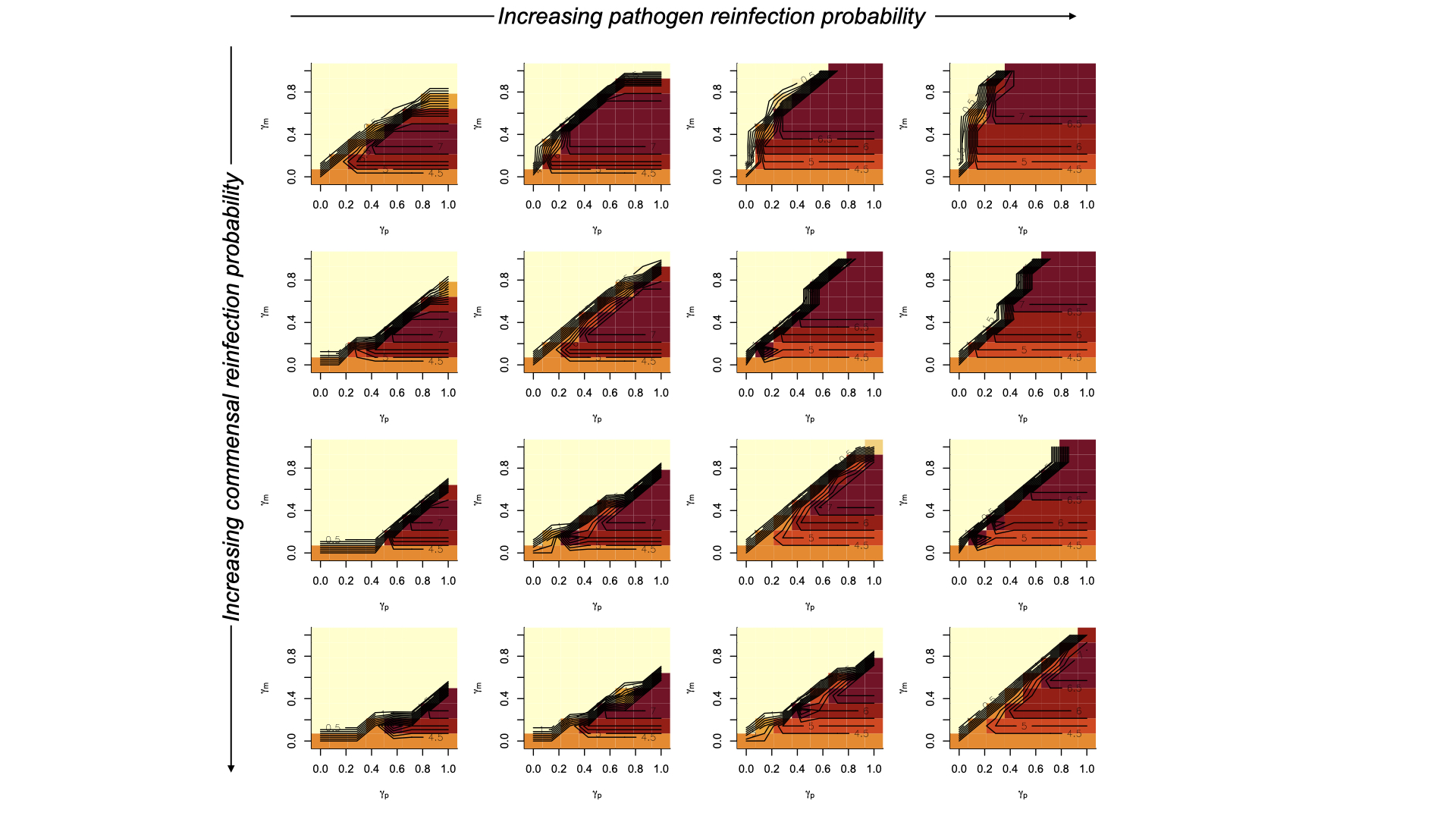

Supplement: Supplementary file 3 — Supplementary figure S1c [file EVL3-6-412-s005.jpeg]

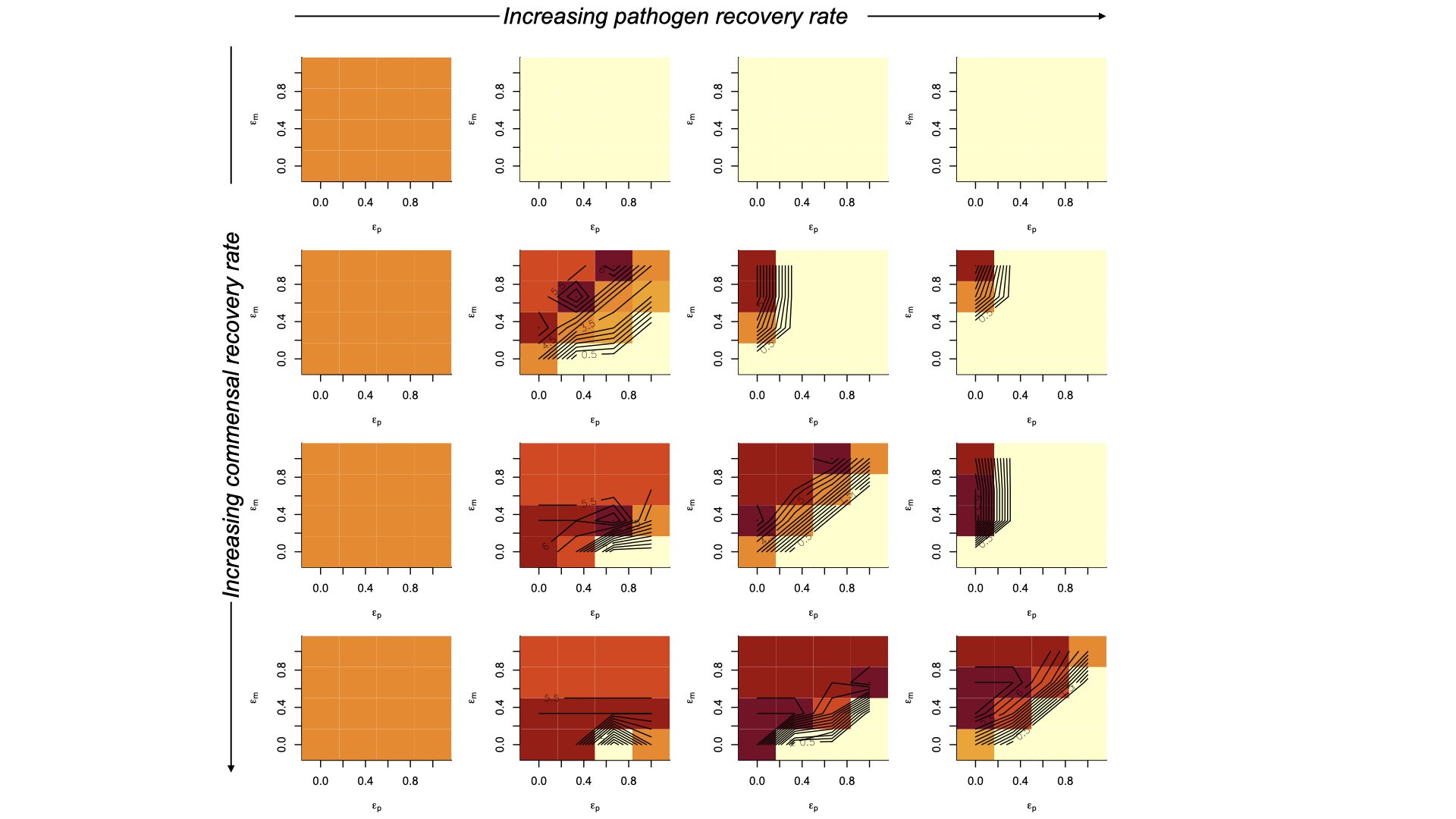

Supplement: Supplementary file 4 — Supplementary figure S1d [file EVL3-6-412-s003.jpeg]
