# Supplementary material for: Hosts, microbiomes, and the evolution of critical windows
Source: Evol Lett. 2022 Oct 27;6(6):412–25. doi: 10.1002/evl3.298 (PMC9783423; doi:10.1002/evl3.298)
Supplement: Supplementary file 5 — Figure S1: The range of optimum windows W* [file EVL3-6-412-s002.pdf]

# Hosts, microbiomes and the evolution of sensitive windows Supplementary Materials: Methods

C. Jessica E. Metcalf, Burcu Tepekule, Marjolein Bruijning, Britt Koskella

To evaluate fitness associated with the length of a critical window, $W$, during which hosts can acquire tolerance to microbes, in a context were both commensal and pathogenic microbes are circulating, we develop a discrete time matrix population model that captures both epidemiological and demographic transitions between age classes and states defined by infection with different types of microbes. Here, we first introduce the structure of the population, then describe how the epidemiological transitions are modeled, and then detail how the demographic transitions are modeled. We provide one illustration of convergence of fitness estimates for two different lengths of the critical window $W$, and detailing the set of steps associated with estimating fitness. All code in R is also available as Supplementary Material.

## 1 Defining the population structure

We characterize host populations according to epidemiological and demographic states via a vector $\mathbf{n}$, encompassing 9 epidemiological states that are also cross-classified by age class. The set of epidemiological states includes $S$, which denotes 'susceptible' individuals (i.e., not and never having been infected by any microbe), $M$, which denotes individuals infected by commensal microbes, $P$, which denotes individuals infected by the pathogenic microbe, and $MP$, which denotes individuals infected by both. The latter three states may also have acquired tolerance by experiencing infection by any type of microbe during the critical window, indicated by the prefix $h$ ($hM$, $hP$, $hMP$, $hR$). For one age class, $a$, we thus have nine epidemiological states ($S_a$, $P_a$, $M_a$, $MP_a$, $R_a$, $hP_a$, $hM_a$, $hMP_a$, $hR_a$), and taking all age classes, the population vector is defined by:

$$\mathbf{n} = \begin{bmatrix} S_1 \\ P_1 \\ M_1 \\ MP_1 \\ R_1 \\ hP_1 \\ hM_1 \\ hMP_1 \\ hR_1 \\ S_2 \\ P_2 \\ M_2 \\ MP_2 \\ R_2 \\ hP_2 \\ hM_2 \\ hMP_2 \\ hR_1 \\ ... \\ ... \\ hP_T \\ hM_T \\ hMP_T \\ hR_T \end{bmatrix}$$

where the subscripts indicates age classes $a = 1$, $a = 2$, etc up to $a = T$, where $T$ is the total number of age classes modeled. The total number of rows in the vector is thus $9 \times T$. To iterate this population forward in time, we need to describe all possible epidemiological and demographic transitions. We address each in turn.

# 2 Epidemiological transitions

We used a discrete-time matrix model to first describe transitions between the possible epidemiological states. First, ignoring transitions associated with aging, birth or mortality, we can write that transitions between epidemiological classes within age class $a$ ordered as above ($S_a$, $P_a$, $M_a$, $MP_a$, $R_a$, $hP_a$, $hM_a$, $hMP_a$, $hR_a$) are captured by the matrix:

$$\mathbf{A}_a = \begin{bmatrix} (1-\phi_p)(1-\phi_m) & 0 & 0 & 0 & 0 & 0 & 0 & 0 & 0 \\ \phi_p(1-\phi_m) & (1-\gamma_p) & 0 & (1-\gamma_p)\gamma_m & \epsilon_p\phi_p(1-\epsilon_m\phi_m) & 0 & 0 & 0 & 0 \\ \phi_m(1-\phi_p) & 0 & (1-\gamma_m) & (1-\gamma_m)\gamma_p & \epsilon_m\phi_m(1-\epsilon_p\phi_p) & 0 & 0 & 0 & 0 \\ \phi_m\phi_p & 0 & 0 & (1-\gamma_p)(1-\gamma_m) & \epsilon_m\phi_m\epsilon_p\phi_p & 0 & 0 & 0 & 0 \\ 0 & \gamma_p & \gamma_m & \gamma_p\gamma_m & (1-\epsilon_p\phi_p)(1-\epsilon_m\phi_m) & 0 & 0 & 0 & 0 \\ 0 & 0 & 0 & 0 & 0 & (1-\gamma_p) & 0 & (1-\gamma_p)\gamma_m & \epsilon_p\phi_p(1-\epsilon_m\phi_m) \\ 0 & 0 & 0 & 0 & 0 & 0 & (1-\gamma_m) & (1-\gamma_m)\gamma_p & \epsilon_m\phi_m(1-\epsilon_p\phi_p) \\ 0 & 0 & 0 & 0 & 0 & 0 & 0 & (1-\gamma_p)(1-\gamma_m) & \epsilon_m\phi_m\epsilon_p\phi_p \\ 0 & 0 & 0 & 0 & 0 & \gamma_p & \gamma_m & \gamma_p\gamma_m & (1-\epsilon_p\phi_p)(1-\epsilon_m\phi_m) \end{bmatrix}$$

where columns capture the epidemiological stage that individuals start in, and rows indicate the epidemiological stage that they go to. Thus, for example, column 1 row 1 indicates individuals staying susceptible, column 1 row 2 indicates individuals moving from susceptible to being infected with the pathogenic microbe, column 1, row 3 indicates individuals moving from being susceptible to being infected with the commensal microbe, and so forth. Transitions in this matrix are defined by **probabilities of infection**, $\phi_p$ and $\phi_m$, the **probabilities of recovery from infection**, $\gamma_p$ and and $\gamma_m$, and the **degree to which probabilities of infection are modulated subsequent to secondary infection**, $\epsilon_p$ and $\epsilon_m$. The latter parameters are defined directlye (see Table 1), while the probabilities of infection are obtained by taking rates of infection obtained via the product of transmission parameters $\beta_p$ and $\beta_m$ (see Table 1) and numbers of infected individuals (appropriately scaled for doubly infected individuals), i.e., $\beta_p(\sum P + hP + 0.5(MP + hMP))$ for pathogens, and converting these rates into probabilities to reflect the discrete time structure of the model:

$\phi_p = 1 - exp(-\beta_p(\sum P + hP + 0.5(MP + hMP)))$

for the pathogen, and:

$\phi_m = 1 - exp(-\beta_m(\sum M + hM + 0.5(MP + hMP)))$

for the commensal microbe (absence of an age subscript indicates that we are taking the vector over all age classes). This expression assumes that transmission from individuals infected before or after the window is equivalent, and that transmission from the doubly infected individuals is halved (qualitative results are robust to this latter assumption). The resulting matrix of epidemiological transitions $A_a$ captures the full set of transitions occurring within every age class $a$, ignoring aging, fertility and mortality. Note that initial entry into the $hP$, $hM$ etc categories reflecting individuals who have acquired tolerance is only possible via transitions occurring out of the age marking the end of the critical window.

# 3 Demographic transitions: survival and aging

Having defined the full set of possible epidemiological transitions, we must now place them within a demographic matrix to also capture the possible set of demographic transitions. We start by considering aging and mortality. In the expression below, each cell within the matrix reflects a different age class. Within that age class, the epidemiological transitions captured by $\mathbf{A}_a$ defined above can occur.

We define the probability of transitioning between age classes as a result of aging $a_g$, baseline probability of mortality as $\mu_b$, mortality associated with the cost of maintaining the window open as $c$, and $\mu_d$ as context specific probability of mortality associated with i) being infected during the window of tolerization ($\mu_p$), ii) not have acquired tolerance and then infected by the commensal microbe after the age of the end of the critical window ($\mu_m$), iii) or being tolerized and then infected by the pathogen after the age of the critical window ($\mu_{p2}$). To reflect the fact that more virulent pathogens are likely to be consistently virulent across the life-cycle, we set $\mu_{p2} = 1.5\mu_p$.

Setting the age class of the end of the critical window as $W = 3$ for illustration, and the total number of age classes tracked $T = 6$ (meaning that we are assuming no further changes in demography are occurring beyond age class 6), the matrix describing demographic as well as epidemiological transitions is defined by:

$$\mathbf{U} = \begin{bmatrix} (1-a_g)\mathbf{A}_1(1-\mu_b-c) & 0 & 0 & 0 & 0 & 0 \\ a_g\mathbf{A}_1(1-\mu_b-c) & (1-a_g)\mathbf{A}_2(1-\mu_b-c-\mu_d) & 0 & 0 & 0 & 0 \\ 0 & a_g\mathbf{A}_2(1-\mu_b-c-\mu_d) & (1-a_g)\mathbf{A}_3(1-\mu_b-c-\mu_d) & 0 & 0 & 0 \\ 0 & 0 & a_g\mathbf{A}_3^*(1-\mu_b-c-\mu_d) & (1-a_g)\mathbf{A}_4(1-\mu_b-\mu_d) & 0 & 0 \\ 0 & 0 & 0 & a_g\mathbf{A}_4(1-\mu_b-\mu_d) & (1-a_g)\mathbf{A}_5(1-\mu_b-\mu_d) & 0 \\ 0 & 0 & 0 & 0 & a_g\mathbf{A}_5(1-\mu_b-\mu_d) & \mathbf{A}_6(1-\mu_b-\mu_d) \end{bmatrix}$$

where, as above, columns indicate the age class that individuals start in, and rows indicate the age class that they end up in. Mortality terms are constrained to be greater than zero. Epidemiological transitions occurring at the end of the critical window (at W) must be modified to reflect the different fates of individuals who are still susceptible by this age class, and individuals who are not. Specifically, at this age class, individuals who have previously been infected by either the pathogenic or commensal microbes transition into the categories indexed by a prefix $t$ to indicate that they have acquired tolerance ($P$ goes to $hP$, $M$ goes to $hM$, etc, see main text, figure 1). The corresponding epidemiological matrix represents a modified form of $\mathbf{A}_a$, denoted $\mathbf{A}_a^*$ and defined by:

$$\mathbf{A}_a^* = \begin{bmatrix} (1-\phi_p)(1-\phi_m) & 0 & 0 & 0 & 0 & 0 & 0 & 0 & 0 \\ \phi_p(1-\phi_m) & 0 & 0 & 0 & 0 & 0 & 0 & 0 & 0 \\ \phi_m(1-\phi_p) & 0 & 0 & 0 & 0 & 0 & 0 & 0 & 0 \\ \phi_m\phi_p & 0 & 0 & 0 & 0 & 0 & 0 & 0 & 0 \\ 0 & 0 & 0 & 0 & 0 & 0 & 0 & 0 & 0 \\ 0 & (1-\gamma_p) & 0 & (1-\gamma_p)\gamma_m & \epsilon_p\phi_p(1-\epsilon_m\phi_m) & (1-\gamma_p) & 0 & (1-\gamma_p)\gamma_m & \epsilon_p\phi_p(1-\epsilon_m\phi_m) \\ 0 & 0 & (1-\gamma_m) & (1-\gamma_m)\gamma_p & \epsilon_m\phi_m(1-\epsilon_p\phi_p) & 0 & (1-\gamma_m) & (1-\gamma_m)\gamma_p & \epsilon_m\phi_m(1-\epsilon_p\phi_p) \\ 0 & 0 & 0 & (1-\gamma_p)(1-\gamma_m) & \epsilon_m\phi_m\epsilon_p\phi_p & 0 & 0 & (1-\gamma_p)(1-\gamma_m) & \epsilon_m\phi_m\epsilon_p\phi_p \\ 0 & \gamma_p & \gamma_m & \gamma_p\gamma_m & (1-\epsilon_p\phi_p)(1-\epsilon_m\phi_m) & \gamma_p & \gamma_m & \gamma_p\gamma_m & (1-\epsilon_p\phi_p)(1-\epsilon_m\phi_m) \end{bmatrix}$$

The core difference is that columns 2-5 now move into rows 6-9, but otherwise follow the same pattern as in $\mathbf{A}_a$.

# 4 Demographic transitions: fertility

Having framed aging and mortality, we turn to fertility, a demographic feature that is also structured by epidemiological and age-class associated transitions. Total fertility per individual is defined by the parameter $f$ (which could be age class dependent, but is kept constant here). Within each age class, the proportion of offspring from individuals infected by the pathogen / the commensal microbe that are born infected with the pathogen / the commensal microbe is determined by the associated probabilities of vertical transmission, $v_p$ and $v_m$ respectively:

$$\mathbf{F}_a = \begin{bmatrix} f & (1-v_p)f & (1-v_m)f & (1-0.5(v_m+v_p))f & f & (1-v_p)f & (1-v_m)f & (1-0.5(v_m+v_p))f & f \\ 0 & v_pf & 0 & 0 & 0 & v_pf & 0 & 0 & 0 \\ 0 & 0 & v_mf & 0 & 0 & 0 & v_mf & 0 & 0 \\ 0 & 0 & 0 & 0.5(v_m+v_p) & 0 & 0 & 0 & 0.5(v_m+v_p) & 0 \\ 0 & 0 & 0 & 0 & 0 & 0 & 0 & 0 & 0 \\ 0 & 0 & 0 & 0 & 0 & 0 & 0 & 0 & 0 \\ 0 & 0 & 0 & 0 & 0 & 0 & 0 & 0 & 0 \\ 0 & 0 & 0 & 0 & 0 & 0 & 0 & 0 & 0 \\ 0 & 0 & 0 & 0 & 0 & 0 & 0 & 0 & 0 \end{bmatrix}$$

We make the simplification of assuming that coinfections are inherited with probability set by the average of vertical transmission of the pathogen and commensal microbe ($0.5(v_m + v_p)$). Using an age class of fertility $A_f = 4$ for illustration and the total number of ages tracked $T = 6$, the matrix determining fertility transitions is defined by:

$$\mathbf{F} = \begin{bmatrix} 0 & 0 & 0 & \mathbf{F}_4 & \mathbf{F}_5 & \mathbf{F}_6 \\ 0 & 0 & 0 & 0 & 0 & 0 \\ 0 & 0 & 0 & 0 & 0 & 0 \\ 0 & 0 & 0 & 0 & 0 & 0 \\ 0 & 0 & 0 & 0 & 0 & 0 \\ 0 & 0 & 0 & 0 & 0 & 0 \end{bmatrix}$$

The combined matrices $\mathbf{U}$ and $\mathbf{F}$ provide a complete description of demographic and epidemiological transition occurring the population in one time step. Assuming a starting population structure $\mathbf{n}$ at time $t$, the population structure one time-step in the future $\mathbf{n}_t$ will be defined by:

$$\mathbf{n}_{t+1} = (\mathbf{F} + \mathbf{U})\mathbf{n}_t$$

via matrix multiplication.

# 5 Estimating fitness

To estimate fitness, we initiate the population structure as a vector of length 9x25 to reflect $N_a = 25$ age classes each containing 9 sequential epidemiological classes ($S$, $P$, $M$, $MP$, $R$, $hP$, $hM$, $hMP$, $hR$). We denote this starting population vector $\mathbf{n}_0$, assuming for simplicity one susceptible in each age class, and one individual infected with the pathogen and one individual infected with the commensal microbe also in each age class (results are robust to different assumptions). We then estimate $\phi_m$ and $\phi_p$ as defined above. From this, we construct the appropriate transition matrix $\mathbf{U}$, and also define the appropriate transition matrix $\mathbf{F}$. We obtain the population structure in the next time step $\mathbf{n}_1$ via

$$\mathbf{n}_1 = (\mathbf{F}{+}\mathbf{U})\mathbf{n}_0.$$

and evaluate the difference in one time-step growth rate, $R_t = log(\sum \mathbf{n}_{t+1} / \sum \mathbf{n}_t)$. If $R_t > 10^{-9}$, we assume that equilibrium has not been reached. We rescale the total population to one for numerical tractability, and iterate the population forwards again (rescaling has no effect on dynamics as we are assuming frequency dependent transmission), once again evaluating $R_t$. We repeat this process until $R_t \leq 10^{-9}$, whereupon we assume that equilibrium has been reached. We then estimate fitness $\lambda$ as the dominant eigenvalue of $\mathbf{F} + \mathbf{U}$. The full set of steps for estimating fitness is shown in Box 1. In order to generate fitness landscapes (as shown for example in Figure 1C) these steps are repeated for each possible value of the window (horizontal axis on Figure 1C), and the length of the window corresponding to the highest fitness is identified as the optimal length of the window, $W^*$ (vertical lines and points on Figure 1C).

**Box 1**: set of steps for estimating fitness.

- Define the full set of parameters (Table 1) alongside the total number of age classes, $T$, the age class corresponding to the end of the critical window $W$, and the age class corresponding to the start of fertility $A_f$
- Initiate a population vector $\mathbf{n}_0$, with arbitrary numbers of individuals in each category, as long as there is at one least one individual infected by the pathogenic and commensal microbes
- Calculate associated probabilities of infection $\phi_p$ and $\phi_m$
- From this, construct $\mathbf{A}_a$ and $\mathbf{A}_a^*$
- From this, construct $\mathbf{U}$
- Construct $\mathbf{F}$
- Calculate $\mathbf{n}_1 = (\mathbf{F}+\mathbf{U})\mathbf{n}_0$
- Calculate $R_1 = log(\sum \mathbf{n}_1 / \sum \mathbf{n}_0)$
- If $R_1 \leq 10^{-9}$, estimate fitness as the dominant eigenvalue of $\mathbf{F} + \mathbf{U}$, and stop here
- If $R_1 > 10^{-9}$, rescale the population vector as $\mathbf{n}_1^{(r)} = \mathbf{n}_1 / \sum \mathbf{n}_1$ (where the $(r)$ indicates rescaling).
- Calculate associated probabilities of infection $\phi_p$ and $\phi_m$
- Calculate $\mathbf{n}_2 = (\mathbf{F}+\mathbf{U})\mathbf{n}_1^{(r)}$
- Calculate $R_2 = log(\sum \mathbf{n}_2 / \sum \mathbf{n}_1^{(r)})$
- If $R_2 \leq 10^{-9}$, estimate fitness; alternatively rescale $\mathbf{n}_2$, ...
- Repeat until $R_t \leq 1e^{-9}$

# 6 Including seasonality

To encompass seasonality in transmission, we include a multiplier of transmission at every time-step defined by $\beta_s = (1 + \alpha cos(2\pi t/24))$ where dividing by 24 indicates the assumption that 24 time-steps make up a year, $\alpha = 0$ corresponds to no seasonality, and $\alpha = 0.7$ corresponds to very strong seasonal forcing (see inset, Figure 5). We assume that seasonality in transmission affects the commensals and the pathogen identically. The force of infection is thus, taking the example of the pathogen, $\phi_p = \beta_s \beta_p (\sum \mathbf{P} + \mathbf{hP} + 0.5(\mathbf{MP} + \mathbf{hMP}))$. Incidence of pathogen and commensals then follow annual cycles. To measure fitness in this fluctuating setting, we take the one time-step growth rate ($R_t = log(\sum \mathbf{n}_{t+1} / \sum \mathbf{n}_t)$) and take the average value of this quantity over the last 100 time-steps of a 1000 time-step simulations. Extending this time-range does not alter results.

# 7 Modeling plasticity

To model plasticity, we extend the epidemiological transition framework $\mathbf{A}_a$ to include four extra rows and columns corresponding to transition for individuals who closed the critical window following early infection by the pathogenic microbe. Epidemiological classes in this model are ordered as for the previously described model, but now extended to include with four extra classes marked by the prefix $p$ to indicate plasticity ($pP_a$, $pM_a$, $pMP_a$, $pR_a$). The resulting $\mathbf{A}_a^p$ matrix includes 13 rows and columns. Individuals in these extra classes are subject to excess immunopathology when infected by the commensal

Figure 1: Example of convergence of the one time-step growth rate $R_t$ for two different lengths, $W$ ($W = 6$ in black and $W = 20$ in red, all other parameters as in Figure 1C of the main text

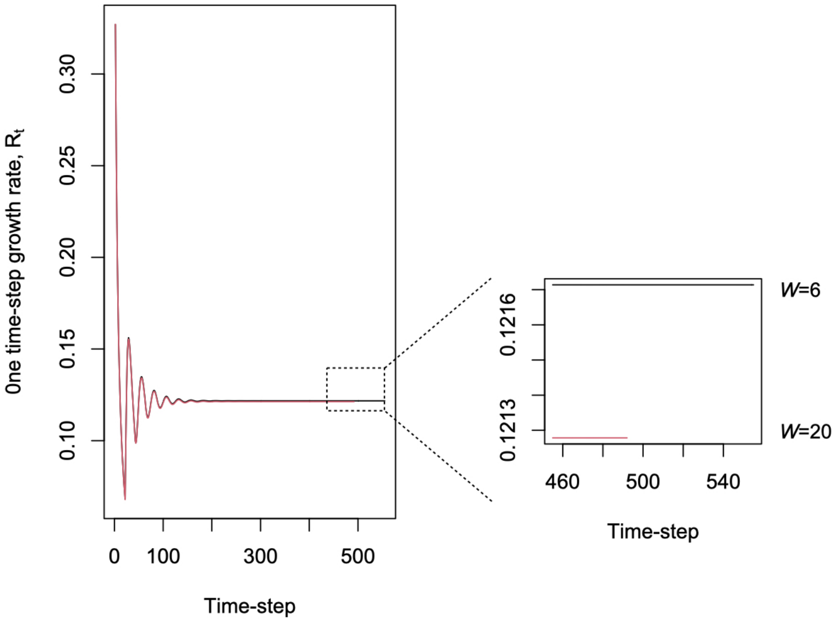


microbe, but do not experience excess mortality associated with pathogenic infection on having acquired tolerance, or the cost of being in the window. To capture individuals entering into this initial stage, the first column of the extended $\mathbf{A}_a$ is modified to be:

$$\mathbf{A}_a^p = \begin{bmatrix} (1-\phi_p)(1-\phi_m) & \dots & \dots \\ 0 & \dots & \dots \\ \phi_m(1-\phi_p) & \dots & \dots \\ 0. & \dots & \dots \\ 0 & \dots & \dots \\ 0 & \dots & \dots \\ 0 & \dots & \dots \\ 0 & \dots & \dots \\ 0 & \dots & \dots \\ \phi_p(1-\phi_m) & \dots & \dots \\ 0 & \dots & \dots \\ \phi_m\phi_p & \dots & \dots \\ 0 & \dots & \dots \end{bmatrix}$$

reflecting the fact that susceptible individuals (captured by the fact that this is the first column) who become singly infected with the pathogen do not move into the usual pathogen infected class (which would be row 2), but instead move down to row 10, which is the new pathogen infected class corresponding to the critical window being close; and similarly for doubly infected individuals who would ordinarily move into row 4. Rows and columns 10 to 13 in $\mathbf{A}_a^p$ then replicate rows and columns 6 to 9 in $\mathbf{A}_a$, but commensal infected individuals experience the excess mortality hazard associated with immunopathology, no individuals experience mortality associated with maintaining the window open, $c$, and there is no mortality associated with being infected by the pathogen having acquired tolerance.
